# Author response to Sinha, “Comment on “Intracranial hemorrhage after ischemic stroke in patients on direct oral anticoagulants: Results from a prospective observational study””

**DOI:** 10.1186/s42466-026-00495-3

**Published:** 2026-06-22

**Authors:** Jan Purrucker, Kirsten Haas, Marilen Sieber, Viktoria Rücker, Peter U. Heuschmann, Roland Veltkamp

**Affiliations:** 1 Department of Neurology, Heidelberg University Hospital, Heidelberg, Germany; 2 Institute of Clinical Epidemiology and Biometry, Julius-Maximilians- Universität Würzburg (JMU), Würzburg, Germany; 3 Clinical Trial Center Würzburg, University Hospital Würzburg, Würzburg, Germany; 4 Institute for Medical Data Science, University Hospital Würzburg, Würzburg, Germany; 5 Department of Neurology, Alfried-Krupp Hospital, Essen, Germany; 6 Department of Brain Research, Imperial College London, Room 10L17, 10th Floor Lab Block, Charing Cross Campus, Margravine Road, London, W6 8RP UK

Dear Editor,

We thank Sinha for the thoughtful comments on our RASUNOA-Prime study [1]. We agree that the issues raised are important for a balanced interpretation of our findings.

As highlighted, patients receiving DOACs presented with less severe strokes than non-anticoagulated patients. Because stroke severity is closely associated with hemorrhagic transformation, this baseline imbalance limits causal interpretation of the lower crude hemorrhage rates observed in the DOAC group. Although we adjusted for relevant confounders, residual confounding cannot be excluded in an observational study, and our results should not be interpreted as demonstrating an intrinsic protective effect of DOAC treatment.

We also agree that between-group differences in reperfusion treatment are highly relevant. Patients without anticoagulation were more likely to receive intravenous thrombolysis [1], whereas anticoagulated patients more often experienced treatment delays. These differences reflect current clinical practice [2, 3], but they also complicate direct group comparisons. In this sense, RASUNOA-Prime should be understood not only as a study of anticoagulation status, but also as a prospective description of how anticoagulation influences treatment selection in routine stroke care.

Sinha also rightly points to the incomplete availability of follow-up imaging. Missing follow-up scans in a substantial proportion of patients introduce uncertainty, particularly for uncommon outcomes such as symptomatic intracranial hemorrhage. Although our sensitivity analyses were directionally consistent with the main results, future studies should aim for protocol-based imaging at predefined time points to reduce ascertainment bias.

We further agree that more systematic assessment of anticoagulant activity would strengthen future research. In our study, laboratory characterization of DOAC activity was available only in a minority of patients, reflecting the practical limitations of emergency stroke care [4]. Broader availability of rapid and standardized coagulation testing could improve both treatment decisions and the interpretability of prospective studies.

In our view, the main contribution of RASUNOA-Prime is not to settle the question of thrombolysis under DOAC therapy, but to provide prospective multicenter data with blinded imaging assessment showing that symptomatic intracranial hemorrhage was uncommon in routine care. These findings add prospective observational evidence to an evolving debate, but randomized trials remain essential before firm changes to clinical recommendations can be justified.

## Data Availability

No datasets were generated or analysed during the current study.
